# Peptide Profiling and Biological Activities of 12-Month Ripened Parmigiano Reggiano Cheese

**DOI:** 10.3390/biology9070170

**Published:** 2020-07-16

**Authors:** Lisa Solieri, Andrea Baldaccini, Serena Martini, Aldo Bianchi, Valentina Pizzamiglio, Davide Tagliazucchi

**Affiliations:** 1 Department of Life Sciences, University of Modena and Reggio Emilia, Via Amendola, 2-Pad. Besta, 42100 Reggio Emilia, Italy; lisa.solieri@unimore.it (L.S.); andreabalda94@gmail.com (A.B.); serena.martini@unimore.it (S.M.); 2 Consorzio del Formaggio Parmigiano Reggiano, via J.F. Kennedy 1 8, 42124 Reggio Emilia, Italy; bianchi@parmigianoreggiano.it (A.B.); pizzamiglio@parmigianoreggiano.it (V.P.)

**Keywords:** proteomic, peptidomic, bioactive peptides, fermented food, lactobacilli

## Abstract

Proteolysis degree, biological activities, and water-soluble peptide patterns were evaluated in 12 month-ripened Parmigiano Reggiano (PR) cheeses collected in different dairy farms and showing different salt and fat content. Samples classified in high-salt and high-fat group (HH) generally showed lower proteolysis degree than samples having low-salt and low-fat content (LL). This positive correlation between salt/fat reduction and proteolysis was also confirmed by the analysis of biological activities, as the LL group showed higher average values of angiotensin-converting enzyme (ACE)-inhibitory and antioxidant activities. UHPLC/HR-MS allowed the identification of 805 unique peptides: LL and HH groups shared 59.3% of these peptides, while 20.9% and 19.9% were LL and HH specific, respectively. Frequency analysis of peptides identified a core of 183 peptides typical of 12-month ripened PR cheeses (corresponding to the 22.7% of total peptides), but no significant differences were detected in peptide patterns between LL and HH groups. Forty bioactive peptides, including 18 ACE-inhibitors and 12 anti-microbial peptides, were identified, of which 25 firstly found in PR cheese. Globally, this work contributed to unraveling the potentially healthy benefits of peptides fraction in PR cheese and provided prior evidence that PR with reduced fat/salt content showed the highest antihypertensive and antioxidant activities.

## 1. Introduction

Several recent review papers have suggested that the intake of dairy foods may be relevant to human health. Total dairy intake is related to a decreased risk of cardiovascular pathologies as well as stroke, hypertension, and colorectal cancer [1]. Furthermore, prospective cohort studies have pointed out a modest association between lower risk of type 2 diabetes and the intake of dairy foods, which is stronger for yogurt [2].

Among dairy products, fermented dairy foods, such as cheese, fermented milk, and yogurt, are getting popularity worldwide for their supposed health-promoting effects [3]. In particular, cheese is not only a plentiful source of fundamental nutrients, such as minerals, vitamins, fats, and proteins, but also of bioactive compounds, mainly fatty acids, calcium, and bioactive peptides, with supposed positive effects on human health [4]. During cheese manufacturing and ripening, milk caseins are hydrolyzed into a broad range of peptides by chymosin, endogenous plasmin, and somatic cells proteolytic enzymes, as well as by starter (S-LAB) and nonstarter (NS-LAB) lactic acid bacteria cell-envelope proteases (CEPs) and peptidases [5,6]. Some of the released peptides, identified in several kinds of cheese, have a proven in vivo or in vitro biological activity, especially anti-microbial, antioxidant, antihypertensive, and ACE-inhibitory activities [3,7,8].

PR is a hard cheese with protected designation of origin (PDO), made by a combination of partially skimmed and whole raw milk added with natural whey starter, principally made up by thermophilic S-LAB [9]. After cooking and brine salting, PR is subjected to at least 12 months of ripening, during which NS-LAB progressively replaces the S-LAB population [10]. The production and maturation procedures of the PR cheese, according to the PDO, are detailed in Tagliazucchi et al. [11]. Reduction of salt and fat intake is a major topic of public health relevance. It is a fundamental nutritional recommendation for reducing the onset of cardiovascular diseases and related pathologies [12,13]. Considering the overall increasing trend in cheese consumption worldwide, the reduction of salt and fat concentrations has become an essential task for public health as well as for the dairy sector [14]. However, lowering fat and/or salt content in cheese may have several negative effects, including altered composition and reduced extent of glycolysis, proteolysis, and lipolysis, with an overall decrease in quality, sensorial attributes, and texture of the final products [15,16,17]. In particular, reduction of fat and salt in cheese increases pH, water activity, and moisture content, as well as decreases salt/moisture (S/M) ratio, which, in turn, leads to the development of bitter taste [18] and to negative alterations of cheese fracture force and firmness [19]. Low S/M levels negatively affect the autolysis of cell permeabilization, reducing the release of microbial aminopeptidases and lipases, mainly responsible for proteolysis and lipolysis [20,21]. Low-fat content further contributes to reducing the free fatty acids, which are precursors of important flavorful molecules, such as ketones, lactones, alcohols, esters, and aldehydes [18]. Fat and salt content also modulates the growth of S-LAB and NS-LAB in cheeses. For example, in Cheddar cheese, the populations of NS-LAB decreases with fat content, whereas the S-LAB population is not affected [22]. PR cheese exhibits a strong variability in salt and fat content, mainly related to practical operations associated with specific cheese-making technologies. This compositional variability modulates the NS-LAB population in PR after 12 months of ripening. *Lactobacillus rhamnosus* is prevalent in PR samples with high-fat and high-salt content, whereas *Lactobacillus paracasei* is most frequent in PR samples with low-fat and low-salt content [11]. Therefore, salt content has a thorough effect on cheese ripening by tuning the bacterial population and thus their proteolytic activity, thereby possibly influencing casein hydrolysis and the subsequent release of peptides and amino acids [22,23]. In this context, the reduction of fat and salt content might modulate the peptidomic profile and, consequently, the bioactive characters of cheeses. Despite the importance of this topic, no studies have investigated the effect of salt and fat content on the release of the peptides during PR cheese ripening.

In our previous work, 24 Parmigiano Reggiano (PR) samples at 12 months of ripening were clustered in two subgroups, namely, LL (samples with low-fat and low-salt content) and HH (samples with high-fat and high-salt content), depending upon their fat and salt content and microbiologically investigated [11]. The present study was designed to compare the peptidome of these two sets of 12-month ripened PR cheese and determined how compositional differences in fat and salt content affected peptide fraction and the associated potential biological activities (angiotensin-converting enzyme inhibitory, dipeptidyl-peptidase IV inhibitory, and antioxidant activities).

## 2. Materials and Methods

### 2.1. Materials

Enzymes, substrates, and reagents for the degree determination of the analysis and hydrolysis of biological activities were supplied by Sigma-Aldrich (Milan, Italy). Mass spectrometry solvents were from Bio-Rad (Hercules, CA, U.S.A.). Amicon Ultra-4 regenerated cellulose filters with a molecular weight cut-off of 3 kDa were purchased from Millipore (Milan, Italy). VPP and IPP (95% purity) were synthesized by Bio-Fab research (Rome, Italy). All the other reagents were from Carlo Erba (Milan, Italy). Parmigiano Reggiano cheese samples were kindly provided by the Consorzio del Formaggio Parmigiano Reggiano (Reggio Emilia, Italy). Cheese wheels produced 12 different dairy farms located in Italy in the provinces of Reggio Emilia (44°42′34″56 N; 10°37′13″80 E), Modena (44°39′24″48 N; 10°55′12″72 E), and Parma (44°48′05.3″ N; 10°19′40.8″ E) from August to September 2017. Two slices of 0.5 kg (height 10.5–11.5 cm, radius 20–24 cm) were collected for each cheese wheel in September 2018 and immediately grated and stored at 4 °C under vacuum for the subsequent biological activities’ analysis and peptide profiling.

### 2.2. Extraction of Water-Soluble Peptides from 12-Month Ripened Parmigiano Reggiano (PR) Samples and Determination of the Peptides Concentration

The extraction of water-soluble peptides from PR was carried out, as reported in Sforza et al. [6], with few modifications. Briefly, 5 g of grated PR samples were combined with 45 mL of 0.1 mol/L HCl and then homogenized with an Ultra-Turrax homogenizer (3 cycles of 1 min). The obtained homogenates were centrifuged for 40 min at 4000× *g* (4 °C). After centrifugation, the supernatants were withdrawn, and the pH brought to 7.0, with NaOH 1 mol/L, to precipitate not hydrolyzed caseins. The peptide fractions were obtained after centrifugation at 10,000× *g* for 20 min at 4 °C. The extractions were performed in triplicate for each PR sample. The TNBS (2,4,6-trinitrobenzenesulfonic acid) assay was applied to quantify the total amount of peptides [24]. The results were expressed as mg of leucine equivalent/g of cheese.

### 2.3. Biological Activities Analysis

#### 2.3.1. Antioxidant Activity

The ABTS (2,2′-azino-bis(3-ethylbenzothiazoline-6-sulphonic acid)) was used to determine the antioxidant activity of the peptide fractions, as described in Re et al. [25]. The ABTS scavenging capacity was expressed as µmol of trolox equivalent/g of cheese.

#### 2.3.2. Angiotensin-Converting Enzyme Inhibitory Activity

Angiotensin-converting enzyme inhibitory activity was measured according to Rutella, Tagliazucchi, and Solieri [26]. Briefly, 350 µL of the tripeptide N-[3-(2-furyl)acryloyl]-L-phenylalanyl-glycyl-glycine (FAPGG) (1.6 mmol/L) dissolved in 100 mmol/L Tris-HCl, buffer containing NaCl 0.6 mol/L (pH 8.2), was mixed with 280 µL of the Tris-HCl buffer and 50 µL of the sample (50 µL of reaction buffer in the control assay). After 3 min of incubation at 37 °C, 20 µL of the ACE solution was added (final ACE activity in the assay of 50 mU/mL). The reaction was followed at 345 nm for 10 min. Results were expressed as a percentage of ACE-inhibitory activity. When possible, the IC_50_ value was calculated by plotting the percentage of ACE inhibition as a function of final sample concentration (base-10 logarithm). IC_50_ is defined as the sample concentration expressed as mg cheese/mL of the test solution able to inhibit the ACE activity by 50%.

#### 2.3.3. Dipeptidyl-Peptidase-IV Inhibitory Activity

The dipeptidyl-peptidase IV (DPP-IV) inhibitory activity was determined, as reported in Tagliazucchi, Martini, Shamsia, Helal, and Conte [27], by using glycine-proline-*p*-nitroanilide (Gly-Pro-pNA) as substrate. Briefly, 100 µL of peptide fraction was mixed in a 96-well plate with 135 µL of 0.1 mol/L Tris-HCl buffer (pH 7.0) and 10 µL of DPP-IV solution (0.1 U/mL). After that, 5 µL of Gly-Pro-*p*NA (6.4 mmol/L) was added, and the reaction mixture was incubated at 37 °C for 20 min. The absorbance was assessed at 405 nm using a microplate reader. Data were reported as the percentage of inhibition of DPP-IV activity.

### 2.4. Peptide Profiling by Ultra-High-Performance Liquid Chromatography/High-Resolution Mass Spectrometry (UHPLC/HR-MS)

The peptide fractions were subjected to UHPLC separation (UHPLC Ultimate 3000, Thermo Scientific, San Jose, CA, USA), followed by high-resolution mass spectrometry (Q Exactive Hybrid Quadrupole-Orbitrap Mass Spectrometer, Thermo Scientific, San Jose, CA, USA) analysis for peptide profiling. Chromatographic separation was performed by using a C18 column (Zorbax SB-C18 Reversed-phase, 2.1 × 50 mm, 1.8 µm particle size, Agilent Technologies, Santa Clara, CA, USA). The mobile phases, the elution gradient, and the mass spectrometry parameters are described in detail in Martini, Conte, and Tagliazucchi [28].

MASCOT (Matrix Science, Boston, MA, USA) protein identification software was utilized for peptide identification. The parameters used for MASCOT analysis were an enzyme, none; peptide mass tolerance, ±5 ppm; fragment mass tolerance, ±0.12 Da; variable modification, oxidation (M) and phosphorylation (ST); the maximal number of post-translational modifications permitted in a single peptide, 4. The identification was confirmed by the manual inspection of fragmentation spectra.

### 2.5. Identification of Bioactive Peptides

Milk bioactive peptides database (MBPDB) was used for the identification of bioactive peptides in the peptide fractions [29]. Peptides with 100% sequence homology to previously described peptides with biological activity were included. Extracted ion chromatograms (EIC) were obtained with a tolerance of ±5 ppm for each peptide. The integration of the AUP (area under the peak) was carried out to obtain the semi-quantitative data. Results were reported as AUP/g of cheese.

### 2.6. Quantification of VPP and IPP by Parallel Reaction Monitoring (PRM)

The lactotripeptides VPP and IPP were quantified, as described in Martini et al., through a standard addition method [28]. The standards linear range was from 0 to 32 µg/L (final concentrations in the samples). The same UHPLC mass spectrometry instrument described above was utilized for the analysis. Each sample (10 µL; 100-fold diluted) was injected two times. The mobile phases, the elution gradient, and the mass spectrometry parameters were accurately described in detail in Martini et al. [23]. The precursor ions selected for IPP and VPP were [M + H]^+^ 326.2074 and *m/z* 312.1918. The product ion y_2_^+^ at *m/z* 213.1234 was selected for the quantitation of VPP and IPP.

The genesis algorithm function in the Thermo Xcalibur Quantitative Browser was utilized for peaks integration. A mass tolerance of 5 ppm was employed for the extraction of target product ions. Calibration curves were built for each analyzed sample, and the peptide concentration in the sample was calculated by determining the x-axis intercept that corresponded to the peptide concentration in the sample.

### 2.7. Statistical Analysis

All data were provided as mean ± standard deviation (SD) for three replicates for each prepared peptide fraction. Univariate analysis of variance (ANOVA) with Tukey post hoc test was applied using GraphPad Prism 6.0 (GraphPad Software, San Diego, CA, USA). The differences were considered significant with *p* < 0.05. Principal component analysis (PCA) was performed using the software package Solo (v. 8.6.1, 2018 Eigenvector Research, Inc., Manson, WA, USA), considering peptides and biological activities as variables.

## 3. Results

### 3.1. Total Peptides Quantification in the Peptide Fractions of 12-Month Ripened Parmigiano Reggiano Samples

**Figure 1:**
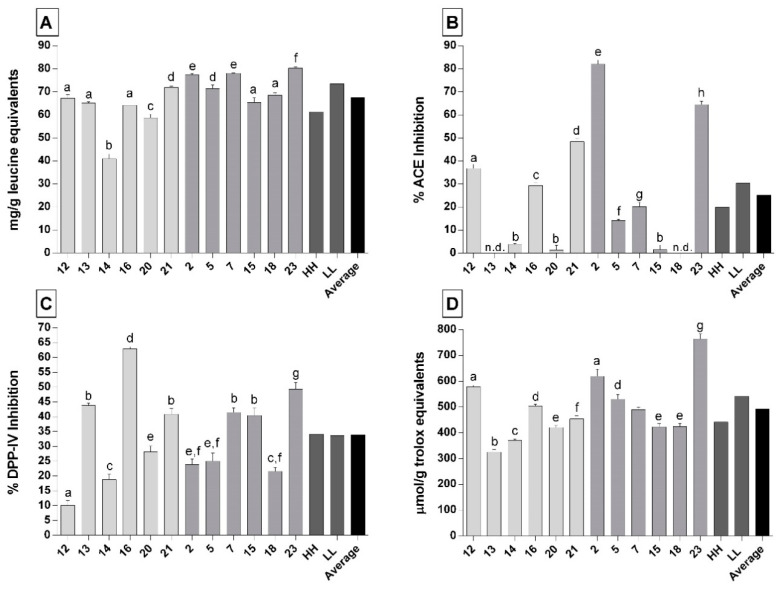
Total peptides concentration and biological activities of 12-month ripened Parmigiano-Reggiano (PR) peptide fractions. (**A**) Total peptides concentration. (**B**) Angiotensin-converting enzyme inhibitory activity. (**C**) Dipeptidyl-peptidase IV-inhibitory activity. (**D**) Antioxidant activity. Light grey bars represent PR samples belonging to the high-salt and high-fat group (HH). Dark grey bars represent PR samples belonging to the low-salt and low-fat group (LL). HH represents the average values, considering the samples belonging to the high-salt and high-fat group. LL represents the average values, considering the samples belonging to the low-salt and low-fat group. Average represents the mean values considering all of the 12 PR samples. The x-axis reports the code number of PR samples. Values are means of three assay replications ± standard deviation (SD). Different letters indicate significantly different values (*p* < 0.05).

In the present study, six Parmigiano Reggiano samples belonging to the category of low-salt and low-fat (LL) and six samples with high-salt and high-fat content (HH) were analyzed for their total peptides content by TNBS assay. The average concentration of total peptides found in the 12 PR samples was 67.4 mg of leucine equivalent/g of cheese. The difference in peptide concentration was clear between the two categories (Figure 1A). In the HH group, the peptide concentrations ranged between 41.0 ± 2.1 and 71.8 ± 0.5 mg of leucine equivalent/g of cheese, with an average value of 61.3 mg of leucine equivalent/g of cheese. Instead, in the LL group, the total average peptides concentration was 73.5 mg of leucine equivalent/g of cheese, higher than that found in the HH group. The range of peptide concentration in the sample of the LL group was from 65.4 ± 2.0 to 80.3 ± 0.5 mg of leucine equivalent/g of cheese. The maximal concentration of peptides was found in the LL sample PR23, followed by PR7 and PR2, whereas the HH sample PR14 showed the lowest peptide concentration (Figure 1A).

### 3.2. Biological Activities of the Peptide Fractions of 12-Month Ripened Parmigiano Reggiano Samples

The overall ACE-inhibitory activity of the peptide fractions extracted from HH and LL PR cheeses is shown in Figure 1B. Larger differences in ACE-inhibitory activity were obtained among the individual samples. The highest ACE-inhibitory activity was found for the LL samples PR2 and PR23 (82.0 and 64.5%, respectively). These samples also showed the highest concentration of peptides among all of the 12 analyzed samples. In the HH group, the highest ACE-inhibitory activity was found for the sample PR21 (48.3%), which also displayed the highest peptide concentration among the sample in the HH group. In this group, three samples (PR13, PR14, and PR20) exhibited undetectable or near-to-zero ACE-inhibitory activity. The average ACE-inhibitory activity was different between the two studied groups, with the LL group showing higher average value with respect to the HH group (Figure 1B). Indeed, we found a positive correlation between the ACE-inhibitory activity and the concentration of total peptides (Pearson r = 0.5973; *p* = 0.040). The IC_50_ data were calculated for the samples with the highest inhibitory activity, resulting in values of 2.2 ± 0.2 and 1.7 ± 0.1 mg of cheese/mL of test solution for sample PR2 and PR23, respectively.

Considerable differences in the DPP-IV-inhibitory activity were also found among samples (Figure 1C). The DPP-IV-inhibitory activity ranged from 10% to 62.9% in the PR12 and PR16 samples, respectively. The average DPP-IV-inhibitory activity values in the LL and HH groups were not different (33.6% and 34.1%, respectively) (Figure 1C).

Antioxidant activity determined with the ABTS assay ranged between 324.7 ± 10.6 and 763.5 ± 19.5 µmol of trolox equivalent/g of cheese in the HH sample PR13 and the LL sample PR23, respectively (Figure 1D). The average value of antioxidant activity in the LL group was significantly higher than that calculated for the HH group (541.4 and 441.9 µmol of trolox equivalent/g of cheese, respectively). Finally, we found a positive correlation between the antioxidant activity and the number of total peptides (Pearson r = 0.6470; *p* = 0.023).

### 3.3. Peptidomic Profile of the Peptide Fractions of 12-Month Ripened Parmigiano Reggiano Samples

Overall, 805 individual peptides were identified in the 12 peptide fractions extracted from 12-month ripened PR cheeses (Table S1). The amount of total identified peptides in the 12 PR samples ranged between 273 and 424 peptides (Figure S1). With the exception of sample PR21, the majority of the peptides originated from β-casein, followed by αS1-casein and αS2-casein. Few peptides deriving from κ-casein were detected only in six samples, whereas no peptides were found from serum proteins, such as α-lactalbumin and β-lactoglobulin (Figure S1).

The average amount of peptides identified in the HH group was a little higher than that found in the LL group (340 vs. 322 peptides) (Figure S1). However, the distribution of the percentage of peptides originating from a single protein was identical between the two groups (Figure S1).

**Figure 2:**
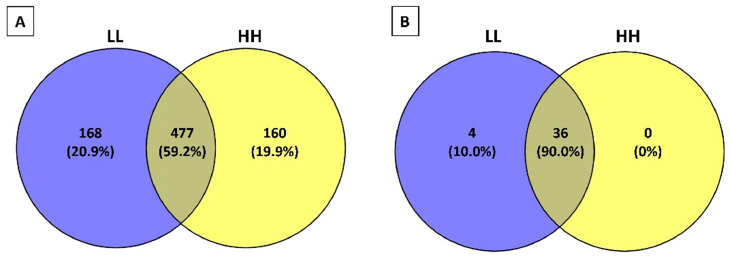
Venn diagrams of peptides obtained from Parmigiano-Reggiano (PR) peptide fractions. (**A**) Venn diagram created with all the identified peptides in the 12 PR peptide fractions (see online supplementary material Table S1 for the peptide sequences). (**B**) Venn diagram created with bioactive peptides identified in the 12 PR peptide fractions (see Table 1 for the peptide sequences). HH represents the samples belonging to the high-salt and high-fat group. LL represents the samples belonging to the low-salt and low-fat group.

The Venn diagram (Figure 2A) showed that 477 peptides (59.3% of total peptides) were commonly found in the PR samples between the LL and HH groups. However, 168 (20.9% of total peptides) and 160 (19.9% of total peptides) peptides were found only in PR samples belonging to the LL group and HH group, respectively. Among the 168 peptides exclusively identified in the LL group PR samples, 44 derived from β-casein, 53 and 52 from αS1-casein and αS2-casein, respectively, and 25 from κ-casein. A similar distribution between proteins was observed among the 160 peptides uniquely found in HH group PR samples, with the majority of the peptides originated from αS1-casein, β-casein, and αS2-casein (56, 55, and 46 peptides, respectively).

**Figure 3:**
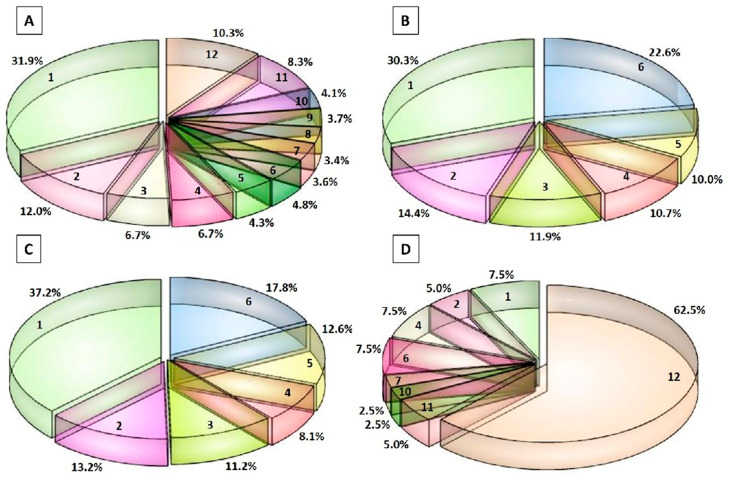
Frequency of identification of the peptides in the Parmigiano-Reggiano (PR) peptide fractions. (**A**) Frequency of identification of the peptides considering all of the 12 PR samples. (**B**) Frequency of identification of the peptides in the 6 PR samples belonging to the high-salt and high-fat group. (**C**) Frequency of identification of the peptides in the 6 PR samples belonging to the low-salt and low-fat group. (**D**) Frequency of identification of the bioactive peptides considering all of the 12 PR samples. The numbers from 1 to 12 or from 1 to 6 indicate the number of PR samples in which the peptides were identified. The percentage values are referred to the % of peptides found in the PR samples. For example, in Figure 3A, 31.9% of single peptides were found in an individual PR sample, whereas the 10.3% of peptides were common in all of the 12 PR samples.

Figure 3 shows the frequency of identification of the peptides in the various samples. Considering all of the samples, the majority of the peptides (43.9%, corresponding to 354 peptides) were found only in one or two samples and were, therefore, presumably generated by chance (Figure 3A). However, 183 peptides (corresponding to 22.7% of total peptides) were found in more than 80% of the samples (≥10 samples).

The frequency of identification between the LL and HH groups was quite similar, with the majority of the peptides found in just one sample of each group (30.3% and 37.2% of total peptides in HH and LL group, respectively) (Figure 3B,C). Nevertheless, as observed above, 30.4% and 32.7% of peptides in the group LL and HH, respectively, were found in more than 80% of the samples (≥5 samples).

### 3.4. Bioactive Peptides in 12-Month Ripened Parmigiano Reggiano Peptide Fractions and Quantification of VPP and IPP

Peptides identified in the 12 PR samples were analyzed with the milk bioactive peptide database (MBPDB) to look for the presence of known bioactive peptides. A total of 40 peptides (Table 1) had 100% sequence homology with bioactive peptides already reported in the literature. The Venn diagram (Figure 2B) shows that 36 bioactive peptides (90% of total peptides) were commonly present in the PR peptide fractions between LL and HH groups, whereas four bioactive peptides were only found in the PR samples from the LL group. The majority of the bioactive peptides identified in the PR peptide fractions were ACE-inhibitors (18 peptides) and anti-microbial (12 peptides). Six bioactive peptides were multifunctional bioactive peptides, one was antioxidant, one was anxiolytic, one was a dipeptidyl peptidase-IV (DPP-IV) inhibitor, and one was a caseinophosphopeptide.

Interestingly, 25 bioactive peptides (corresponding to the 62.5% of identified bioactive peptides) were found in all of the 12 PR samples, suggesting that these bioactive peptides were commonly released in 12-month ripened PR cheeses by the action of LAB CEPs and endopeptidases (Figure 3D).

The relative abundance of 36 bioactive peptides was determined and reported in Supplementary Table S2. Larger differences were obtained among the 12 PR samples and among samples within the same group. For only two bioactive peptides, the mean abundance was different between the HH and LL groups (*p* < 0.05). For both the peptides, DKIHPF and YQEPVLGPVRGPFPIIV, the mean abundance was significantly higher in the HH group with respect to the LL group.

As shown in Table 2 and Table 3, VPP and IPP were identified and quantified in the peptide fractions of all the 12 PR samples in amounts ranging from 3.27 ± 0.10 to 16.36 ± 0.96 mg/kg for VPP and from 0.61 ± 0.04 to 2.76 ± 0.17 mg/kg for IPP. The highest concentrations of VPP were found in the HH samples PR13 and PR20 (16.36 ± 0.96 and 10.49 ± 0.74 mg/kg, respectively). Similarly, the highest IPP concentrations were found in the HH samples PR13 and PR14 (2.76 ± 0.17 and 1.64 ± 0.09 mg/kg, respectively). The average amounts of VPP and IPP were similar between the HH and LL groups (Table 2 and Table 3).

### 3.5. Relationship between the ACE-Inhibitory Activity and ACE-Inhibitory Peptides Profile in 12-Month Ripened Parmigiano Reggiano Peptide Fractions

**Figure 4:**
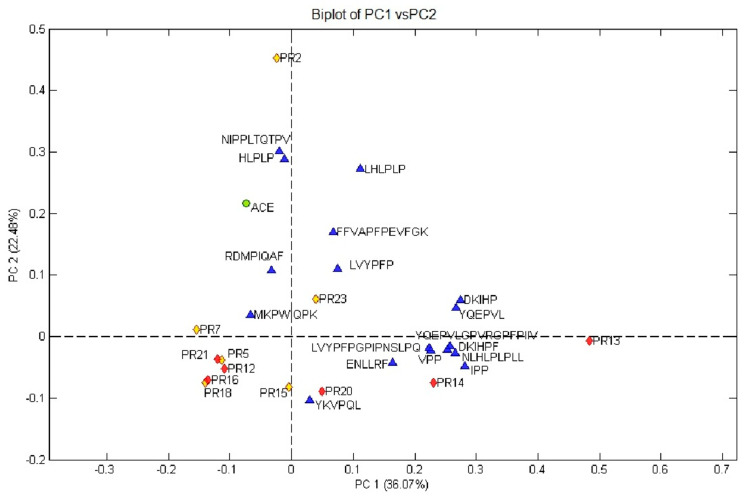
Distribution of peptides characterized by angiotensin-converting enzyme (ACE) inhibitory IC_50_ values lower than 300 µmol/L along with principal components 1 (PC1) and 2 (PC2). IC_50_ represent the concentration of peptide able to inhibit the enzymatic activity by 50%. Yellow diamonds represent LL class samples, red diamonds represent HH class samples, green circle represents the biological activity (ACE-inhibition), and blue triangles represent the bioactive peptides.

Despite the distribution of the identified bioactive peptides in the peptide fractions of the 12 PR samples was quite similar, we found substantial differences in the biological activities among samples, especially regarding the ACE-inhibitory activity. The exploratory analysis of the principal components (PCA) was carried out to obtain a quick comprehension of the data, showing all the possible associations and networks between ACE-inhibitory peptides, samples, and ACE-inhibitory activity. This approach provided information associated with the collection of multivariate data, where peptides and activities might be graphed as a linear combination of orthogonal principal components (PCs). Three principal components explained about 76% of the total variance. The first two components were selected for the construction of the bi-dimensional plot (PC1×PC2 biplot) reported in Figure 4, describing the 58.6% of the total variance cumulative percentage. In particular, the PC1×PC2 biplot indicated an unequivocal separation of PR2 sample on the second component, resulting in a positive correlation with the ACE inhibitory activity. This output plot confirmed the results obtained with the in vitro assays. In order to figure out the responsible peptides for the given distribution and the inhibitory effect, they were added to the bi-dimensional plot. Interestingly, two samples emerged from the PC analysis, displaying a possible peptides-ACE inhibition relation. PR2, positively linked to PC2 and separated from the other samples, was more effective in ACE inhibition, which was positively correlated. As outlined by the positive correlation on PC2 and positive loadings, PR2 featured the highest amount of NLHLPLPLL, HLPLP, and LHLPLP. Hereby, considering their very low IC_50_ values, they could be speculated to be the causative peptides for the ACE inhibitory effect of PR2. Nevertheless, PR23 was the second higher effective for its ACE-inhibition and confirmed by the positive scores on PC2. The positive score on PC1 was related to the presence and concentration of different peptides with respect to those of PR2. In fact, the positive loadings on PC1 of LHLPLP, LVYPFP, and FFVAPFPEVFGK reflected their higher concentration in PR23 than PR2, with the exception of LHLPLP, which was still present in high amount in PR2. These latter peptides, positively linked on PC2, also recording very low IC_50_ values, could be considered responsible for the ACE-inhibitory effect of PR23. Hence, these results might suggest a potential impact of the manufacturer process (LL) in the specific bioactive peptides release and the studied biological effect.

## 4. Discussion

Strong scientific pieces of evidence have suggested that a diet high in fat and salt might result in an increased onset of hypertension and cardiovascular diseases [30,31]. Dietary intervention by reducing the intake of fat and salt has seemed to be the most promising way to decrease the onset of cardiovascular diseases in the population. Dietary salt intake reduction has been associated with a decrease in blood pressure and in the mortality of cardiovascular diseases [30]. Similarly, a decrease in fat intake has been correlated with a reduction in body mass index and weight, decreasing the risk of cardiovascular diseases [31].

Even if several studies have unraveled the effect of salt and fat reduction on the sensory and safety properties of cheese, no studies on the impact of salt and fat reduction on the biological properties and peptidomic profile of cheese have been carried out until now. Therefore, this work was designed to deal with this topic by analyzing the differences in proteolysis, biological activities, and the release of bioactive peptides in PR samples previously divided into two subgroups, namely, high-fat and high-salt (HH) group and low-fat and low-salt (LL) group [11].

Proteolysis is one of the most important biochemical events occurring during cheese processing and ripening [6]. The hydrolysis of milk caseins is initiated by chymosin; proteases already present in milk, such as plasmin, as well as proteases present in somatic cells and psychrotrophic bacteria [6]. Large oligopeptides produced by chymosin and endogenous proteolytic enzymes represent the substrate for proteinases and peptidases existing in S-LAB and NS-LAB [32]. Hydrolysis of caseins and casein-derived large oligopeptides is carried out by LAB CEPs, which are able to release oligopeptides of about 5–30 amino acids length [3]. These peptides can be transported inside the cell, where cytoplasmic peptidases may further hydrolyze them into smaller peptides and free amino acids [3]. Sforza et al. [6] showed that peptides released by the action of chymosin and endogenous proteolytic enzymes dominated the peptide profile of PR cheese at the beginning of the curding process and then were promptly hydrolyzed during the first hours after curding by the action of S-LAB proteinases. After 10 months of ripening, a dramatic alteration in the peptide profile was observed, as NS-LAB became the predominant LAB population present in the PR cheese.

Evaluation of the concentration of free amino groups (a measure of total peptides and amino acids) in cheeses from HH and LL group showed that samples from the LL group contained a higher amount of free amino groups with respect to the samples from the HH group, suggesting a more pronounced proteolytic activity. Congruently, the amount of free amino acids has increased significantly with decreasing fat content in Cheddar cheese [22]. These results also agreed with previous observations in Cheddar cheese, where a decrease in NaCl content has significantly enhanced proteolysis [17,33,34,35]. By contrast, in Prato cheese, NaCl reduction does not affect proteolysis [36]. Increased salt content has enhanced the activity of LAB CEPs and some peptidases, such as PepX and PepI, in *Lactobacillus lactis* [37]. In PR cheese with high-salt content, *L. rhamnosus* is predominant, whereas, in contrast, *L. paracasei* is prevalent in PR cheese with low-salt content [11]. Proteolytic activity analysis has revealed that the *L. rhamnosus* strains isolated from high-salt PR samples have higher hydrolytic ability than the *L. paracasei* strains isolated from PR samples at low-salt content [11]. Intracellular peptide accumulation is considered as an osmo-protective mechanism in LAB during growth in the high-salt medium [37]. Therefore, the lower concentrations of peptides found in HH group PR samples may be related to an increased ability of the NS-LAB population, inhabiting high-salt samples to counter-balance low water activity values through the peptide intake. Alternatively, we can speculate that *L. paracasei* is more prone than *L. rhamnosus* to lysis, releasing intracellular enzymes into the cheese environment.

Biological activity analysis revealed great variation among samples, though the average ACE-inhibitory and antioxidant activities were higher in the LL group than in the HH group. Different factors contributed to the high inter-sample variability observed in this work. Variations in milk quality and content in somatic cells impact cheese-making yield and the extent of proteolysis by indigenous milk proteases [38,39,40]. Another source of variation could be the natural whey starter used in the production of PR cheese, which is a complex association of different LAB species and distinct intra-species biotypes [41,42,43,44]. NS-LAB microbiota represents a further source of variability as distinct NS-LAB fractions have been found in different factories [9,45,46]. This would result in a diverse pattern or amount of released bioactive peptides, which, in turn, may affect the biological activity of the samples. Despite the “noise” effects of these variables, we observed significant inter-group (LL vs. HH) differences in the tested biological activities, which might reflect the effect of different fat and salt concentrations. These two compositional parameters have an impact on the composition of microbial populations and the associated proteolytic activities [11], which modulate the subsequent release of peptides with biological activities.

It is hard to compare the results of the ACE-inhibitory activity of the different studies since different ACE-inhibition assays and extraction methods have been applied. Indeed, the way for reporting IC_50_ values is not always uniform as the data may be a function of total protein content, total peptide content, or amount of cheese. In this study, we reported the IC_50_ data as the mg of cheese in the test solution necessary to obtain an inhibition of the ACE activity of 50%. In this respect, our data were in accordance with that reported by Bütikofer, Meyer, Sieber, and Wechsler [47] for hard and semi-hard cheeses. The results on DPP-IV-inhibitory activity were lower than those previously reported for the peptide fraction of gouda-type cheese [48], whereas antioxidant activity data were comparable to those already reported in the literature for PR and Cheddar cheeses peptide fractions [49,50].

Among the 805 peptides identified in the different samples, 59.2% were commonly found in at least one sample in LL and HH groups. However, only 22.7% of peptides were identified in more than 80% of the sample, suggesting that these peptides were produced by the specific action of LAB proteinase/peptidases and were characteristic and typical of 12-month ripened PR cheeses.

The majority of the 183 peptides typical of 12-month ripened PR cheeses originated from β-casein. Among them, 44 peptides were from the N-terminal part of the protein. Several peptides in this group were from sequence 1–29 of β-casein. Most of these peptides originated from the specific action of LAB CEPs, as indicated by the typical CEPs cleavage site L_6_-N_7_, N_14_-K_15_, E_21_-S_22_, N_27_-K_28_, and K_29_-I_30_ [49,50,51]. It is important to note that the peptide bond K_28_-K_29_ is a preferential cleavage site for milk plasmin [51].

Only 16 peptides were found from the C-terminus of β-casein. It is well known that LAB CEPs hydrolyze preferentially the C-terminus of β-casein [52]. Therefore, it was possible that peptides released from the C-terminus were hydrolyzed to shorter peptides by the action of LAB CEPs and LAB endopeptidases. Despite peptide Y_193_-V_209_ can be originated by the action of chymosin [6], most of the C-terminus peptides derived from CEPs hydrolysis, as depicted by the numerous characteristic CEPs cleavage sites [52,53,54].

The principal chymosin cleavage site in αS1-casein is the peptide bond F_23_-F_24_ releasing the peptide R_1_-F_23_ [6]. This peptide was also detected in the present study as typical of 12-month ripened PR cheese together with additional four peptides derived from the action of LAB CEPS and LAB endopeptidases on peptide R_1_-F_23_. Further peptides (F_24_-K_34_, F_24_-K_36_, and F_24_-E_38_) detected at the beginning of the curding in PR presumably released by the action of chymosin were also found as typical of 12-month ripened PR cheese [6]. Several peptides deriving from the CEPs action on these peptides were also identified in more than 80% of the samples.

Among the 40 identified bioactive peptides, 15 have been already reported in Parmigiano Reggiano cheeses at different ripening times [28], whereas the remaining 25 bioactive peptides were reported for the first time in this study. A total of 13 peptides were ACE-inhibitors, and most of them have been already found in cheese or other fermented dairy products [3]. Some of these identified peptides have been previously reported as able to decrease blood pressure in vivo [3]. The β-casein-derived peptide KVLPVPQ, previously isolated from a commercial functional yogurt, has shown strong antihypertensive effects (−31.5 mmHg) at a dosage of 2 mg/kg in spontaneously hypertensive rats [55,56]. The peptide LHLPLP, previously identified in various cheeses (such as Grana Padano, Parmigiano Reggiano, Gorgonzola, and Cheddar), exhibited very low IC_50_ value *versus* ACE and has been found able to decrease blood pressure (−25.3 mmHg at a dosage of 3 mg/kg) in spontaneously hypertensive rats [57,58,59,60]. Additional peptides found in all of the 12 PR samples showed potent ACE-inhibitory activity, similar to the peptides NLHLPLPLL and YPFPGPIPN, both showing IC_50_ value of 15 µmol/L, and the peptide YQEPVL, displaying an IC_50_ value of 8 µmol/L [61,62,63]. Some other peptides exhibited a high inhibitory effect against ACE and in vivo antihypertensive effect. The αS1-casein-derived peptide YKVPQL was detected in 11 out of 12 PR samples and has been previously identified as an in vitro and in vivo antihypertensive peptide [59]. Similarly, also the β-casein-derived peptide HLPLP has exhibited in vitro and in vivo antihypertensive effect, but it was identified only in one sample (PR2) belonging to the LL group [64].

The lactotripeptides VPP and IPP, previously reported as antihypertensive molecules in vivo on humans, were quantified in the peptide fractions of the 12 PR samples [65]. The quantitative data were in agreement with the concentrations of VPP and IPP, previously reported in the PR sample at 12 months of ripening [28,57].

Because all of the PR samples used in this study were produced following the PDO disciplinary, which harmonizes and uniforms the processing conditions, it is unlikely that dissimilarity in heating up and ripening phases may account for the substantial variation in the number of bioactive peptides observed among the individual samples. Furthermore, qualitative and semi-quantitative data suggested that differences in salt and fat content only partially affected the bioactive peptides profile of PR samples. Indeed, previous studies have suggested that raw milk used for PR cheese-making [66], as well as S-LAB used for cheese processing, has a preponderant effect on the ACE-inhibitory properties and bioactive peptides production during cheese ripening [67,68]. Therefore, it is likely that the differences found in this study among the 12 PR samples were a consequence of distinct populations of S-LAB and NS-LAB during cheese-making and ripening, respectively.

## 5. Conclusions

The Production Specification regulated by the Consortium of PR cheese governs the PR cheese-making procedures, providing general guidelines for brining and natural skimming of evening milk, the two main steps that affect salt and fat contents. In particular, it established the fat/casein ratio in vat milk lower than 1.1. Therefore, strong inter-dairy operational variability accounts for variation in salt and fat content in PR cheese. In this study, we first considered how these differences in salt and fat levels might affect the proteolytic degree and biological activities in PR samples after 12 months of ripening, as well as their content in the bioactive peptide. We delineated the general trend that low-salt and low-fat contents positively affected proteolysis and biological activities contents in PR cheese, whereas they did not negatively affect the content in bioactive peptides, which exhibited a dairy-specific pattern. Our results provided a framework on the impact of salt and fat content on the proteolytic activity and on the biological effects, taking into account all the possible existing variables (such as milk batches, S-LAB cultures, etc.) attending the PR manufacturing process. Further investigations are required to establish the extent of these salt/fat reductions and the impact on other technological and organoleptic parameters also during further stages of ripening. This pilot work might pave the way for future tailored standard dairy processes basing only on the salt and fat content variation. The data collected here sketched out possible tailoring to boost the cheese-making technological process in tune with WHO’s guidelines on consumer’s health.

## Figures and Tables

**Table 1:** List of the bioactive peptides found in the peptide fractions of Parmigiano Reggiano (PR) samples at 12 months of ripening ^a^.

Sequence ^b^	Fragment	Activity ^c^	LL Samples	HH Samples
**β-casein**				
RELEELNVPGEIVESLSSSEESITR	1–25	Caseinophosphopeptide	PR2, PR5, PR7, PR15, PR18, PR23	PR14
TEDELQDKIHPF	41–52	Anti-microbial	PR2, PR5, PR7, PR15, PR18, PR23	PR12, PR13, PR14, PR16, PR20, PR21
DKIHP	47–51	ACE-inhibition (IC_50_ = 113 µmol/L)	PR2, PR5, PR7, PR15, PR18, PR23	PR12, PR13, PR14, PR16, PR20, PR21
DKIHPF	47–52	ACE-inhibition (IC_50_ = 257 µmol/L)	PR2, PR5, PR7, PR15, PR18, PR23	PR12, PR13, PR14, PR16, PR20, PR21
LVYPFP	58–63	ACE-inhibition (IC_50_ = 132 µmol/L)	PR2, PR5, PR7, PR15, PR18, PR23	PR12, PR13, PR14, PR16, PR20, PR21
LVYPFPGPIPNSLPQ	58–72	ACE-inhibition (IC_50_ = 18 µmol/L)	PR2, PR5, PR7, PR15, PR18, PR23	PR12, PR13, PR14, PR16, PR20, PR21
VYPFPGPIPN	59–68	ACE-inhibition (IC_50_ = 325 µmol/L) Antihypertensive (−7.0 mmHg) Antioxidant	PR2, PR5, PR7, PR15, PR18, PR23	PR12, PR13, PR14, PR16, PR20, PR21
YPFPGPIPN	60–68	ACE-inhibition (IC_50_ = 15 µmol/L) Antihypertensive (−7.0 mmHg) DPP-IV-inhibition (IC_50_ = 670 µmol/L)	PR2, PR5, PR7, PR15, PR18, PR23	PR12, PR13, PR14, PR16, PR20, PR21
PGPIPN	63–68	Immunomodulatory Anti-cancer	PR2, PR5, PR7, PR15, PR18, PR23	PR12, PR13, PR14, PR16, PR20, PR21
NIPPLTQTPV	73–82	ACE-inhibition (IC_50_ = 173 µmol/L)	PR2	n.d.
TQTPVVVPPFLQPE	78–91	Antioxidant	PR2, PR5, PR7, PR15, PR18, PR23	PR12, PR13, PR14, PR16, PR20, PR21
PVVVPPFLQPE	81–91	Anti-microbial	PR2	n.d.
VKEAMAPK	98–105	Anti-microbial Antioxidant	PR2, PR5, PR7, PR15, PR18, PR23	PR12, PR13, PR14, PR16, PR20, PR21
YPVEPF	114–119	Opioid DPP-IV-inhibition (IC_50_ = 125 µmol/L)	PR2, PR5, PR7, PR15, PR18, PR23	PR12, PR13, PR14, PR16, PR20, PR21
NLHLPLPLL	132–140	ACE-inhibition (IC_50_ = 15 µmol/L)	PR2, PR5, PR7, PR15, PR18, PR23	PR12, PR13, PR14, PR16, PR20, PR21
LHLPLP	133–138	ACE-inhibition (IC_50_ = 4 µmol/L) Antihypertensive (−25.3 mmHg)	PR2, PR5, PR7, PR15, PR18, PR23	PR12, PR13, PR14, PR16, PR20, PR21
HLPLP	134–138	ACE-inhibition (IC_50_ = 41 µmol/L) Antihypertensive (−23.5 mmHg)	PR2	n.d.
KVLPVPQ	159–175	ACE-inhibition (IC_50_ = 1000 µmol/L) Antihypertensive (−31.5 mmHg)	PR2, PR5, PR7, PR15, PR18, PR23	PR12, PR13, PR14, PR16, PR20, PR21
RDMPIQAF	183–190	ACE-inhibition (IC_50_ = 209 µmol/L)	PR2, PR7, PR15, PR23	n.d.
YQEPVLGPVRGPFPI	193–207	Anti-microbial	PR2, PR5, PR7, PR15, PR18, PR23	PR12, PR13, PR14, PR16, PR20, PR21
YQEPVLGPVRGPFPIIV	193–209	ACE-inhibition (IC_50_ = 101 µmol/L) Anti-microbial Antioxidant Immunomodulatory	PR2, PR5, PR7, PR15, PR18, PR23	PR12, PR13, PR14, PR16, PR20, PR21
QEPVLGPVRGPFPIIV	194–209	ACE-inhibition (IC_50_ = 600 µmol/L)	PR2, PR5, PR7, PR15, PR18, PR23	PR12, PR13, PR14, PR16, PR20, PR21
VRGPFPIIV	201–209	ACE-inhibition (IC_50_ = 600 µmol/L)	PR2, PR5, PR7, PR15, PR18, PR23	PR12, PR13, PR14, PR16, PR20, PR21
**αS1-casein**				PR12, PR13, PR14, PR16, PR20, PR21
RPKHPIKHQGLPQEVLNENLLRF	1–23	Anti-microbial	PR2, PR5, PR7, PR15, PR18, PR23	PR12, PR13, PR14, PR16, PR20, PR21
ENLLRF	18–23	ACE-inhibition (IC_50_ = 82 µmol/L)	PR2, PR5, PR7, PR15, PR18, PR23	PR12, PR13, PR14, PR16, PR20, PR21
FFVAPFPEVFGK	23–34	ACE-inhibition (IC_50_ = 52 µmol/L) Antihypertensive (−34.0 mmHg)	PR2, PR5, PR7, PR15, PR18, PR23	PR12, PR13, PR14, PR16, PR20, PR21
HIQKEDVPSERYLGYLEQLLRLK	80–102	Anti-microbial	PR2, PR5, PR7, PR15, PR18, PR23	PR13, PR16, PR20, PR21
HIQKEDVPSERYLGYLEQLLRLKKYK	80–102	Anti-microbial	PR2, PR5, PR7, PR15, PR18, PR23	PR13, PR16, PR20, PR21
YLGYLEQLLR	91–101	Anxiolytic	PR2, PR5, PR7, PR15, PR18, PR23	PR12, PR13, PR14, PR16, PR20, PR21
LRLKKYKVPQL	99–109	Anti-microbial	PR5, PR7, PR15, PR18, PR23	PR12, PR13
YKVPQL	104–109	ACE-inhibition (IC_50_ = 22 µmol/L) Antihypertensive (−12.5 mmHg)	PR5, PR7, PR15, PR18, PR23	PR12, PR13, PR14, PR16, PR20, PR21
αS2-casein				PR12, PR13, PR14, PR16, PR20, PR21
IVLNPWDQVK	104–113	Anti-microbial	PR2	PR14
VPITPT	117–140	DPP-IV-inhibition (IC_50_ = 130 µmol/L)	PR2, PR5, PR7, PR15, PR18, PR23	PR12, PR13, PR14, PR16, PR20, PR21
TVYQHQKAMKPWIQPKTKVIPYVRYL	182–207	Anti-microbial	PR2, PR5, PR7, PR15, PR18, PR23	PR12, PR13, PR14, PR16, PR20, PR21
VYQHQKAMKPWIQPKTKVIPYVRYL	183–207	Anti-microbial	PR2, PR5, PR7, PR15, PR18, PR23	PR12, PR13, PR14, PR16, PR20, PR21
AMKPWIQPK	189–197	ACE-inhibition (IC_50_ = 600 µmol/L)	PR2, PR5, PR7, PR15, PR18, PR23	PR12, PR13, PR14, PR16, PR20, PR21
MKPWIQPK	190–197	ACE-inhibition (IC_50_ = 300 µmol/L)	PR2, PR5, PR7, PR15, PR18, PR23	PR12, PR14, PR16, PR20, PR21
WIQPKTKVIPYVRYL	193–207	Anti-microbial	PR15, PR18	PR13, PR14
TKVIPYVRYL	198–207	Anti-microbial	PR2, PR5, PR7, PR15, PR18, PR23	PR12, PR13, PR14, PR16, PR20, PR21

^a^ Abbreviations: ACE, angiotensin-converting enzyme; DPP-IV, dipeptidyl peptidase-IV. ^b^ One code letter was used for amino acid nomenclature. ^c^ Potential bioactivities were achieved from the milk bioactive peptides database (MBPDB) [24]. IC_50_ represents the concentration of peptide able to inhibit the enzymatic activity by 50%. The antihypertensive activity was measured on spontaneously antihypertensive rats. n.d. means peptide not detected in any sample of the specific LL (low-salt and low-fat content) or HH (high-salt and high-fat content) group.

**Table 2:** Amount of VPP and IPP in the peptide fractions of Parmigiano Reggiano (PR) samples at 12 months of ripening belonging to the group HH (high-fat and high-salt).

Sequence *	PR12 mg/kg	PR13 mg/kg	PR14 mg/kg	PR16 mg/kg	PR20 mg/kg	PR21 mg/kg	Average mg/kg
VPP	3.41 ± 0.18 ^a^	16.36 ± 0.96 ^b^	6.57 ± 0.31 ^c^	3.64 ± 0.11 ^a^	10.49 ± 0.74 ^d^	4.34 ± 0.34 ^e^	7.47
IPP	0.62 ± 0.04 ^a^	2.76 ± 0.17 ^b^	1.64 ± 0.09 ^c^	0.65 ± 0.02 ^a^	0.99 ± 0.05 ^d^	0.63 ± 0.02 ^a^	1.22

* One code letter was used for amino acid nomenclature. Different letters within the same row mean significantly different (*p* < 0.05) values.

**Table 3:** Amount of VPP and IPP in the peptide fractions of Parmigiano Reggiano (PR) samples at 12 months of ripening belonging to the group LL (low-fat and low-salt).

Sequence *	PR2 mg/kg	PR5 mg/kg	PR7 mg/kg	PR15 mg/kg	PR18 mg/kg	PR23 mg/kg	Average mg/kg
VPP	4.84 ± 0.21 ^a^	5.11 ± 0.22 ^a^	5.67 ± 0.29 ^b^	7.90 ± 0.33 ^c^	3.27 ± 0.10 ^d^	6.82 ± 0.31 ^e^	5.60
IPP	0.61 ± 0.04 ^a^	0.86 ± 0.07 ^b^	1.01 ± 0.08 ^b^	1.50 ± 0.09 ^c^	0.70 ± 0.04 ^a^	1.31 ± 0.09 ^c^	1.00

* One code letter was used for amino acid nomenclature. Different letters within the same row mean significantly different (*p* < 0.05) values.

## Supplementary Materials

The following are available online at https://www.mdpi.com/2079-7737/9/7/170/s1, Figure S1: Number of unique peptides identified in the Parmigiano Reggiano samples, Table S1: Peptides identified in the Parmigiano Reggiano samples, Table S2: Relative quantification of bioactive peptides in the Parmigiano Reggiano samples.

## References

[1] Godos J., Tieri M., Ghelfi F., Titta L., Marventano S., Lafranconi A., Gambera A., Alonzo E., Sciacca S., Buscemi S. (2019). Dairy foods and health: An umbrella review of observational studies. Int. J. Food Sci. Nutr..

[2] Guo J., Givens D.I., Astrup A., Bakker S.J.L., Goossens G.H., Kratz M., Marette A., Pijl H., Soedamah-Muthu S.S. (2019). The impact of dairy products in the development of type 2 diabetes: Where does the evidence stand in 2019?. Adv. Nutr..

[3] Tagliazucchi D., Martini S., Solieri L. (2019). Bioprospecting for bioactive peptide production by lactic acid bacteria isolated from fermented dairy food. Fermentation.

[4] Summer A., Formaggioni P., Franceschi P., Di Frangia F., Righi F., Malacarne M. (2017). Cheese as functional food: The example of Parmigiano-Reggiano and Grana Padano. Food Technol. Biotech..

[5] Sforza S., Galaverna G., Neviani E., Pinelli C., Dossena A., Marchelli M. (2004). Study of the oligopeptide fraction in Grana Padano and Parmigiano-Reggiano cheeses by liquid chromatography-electrospray ionization mass spectrometry. Eur. J. Mass Spectrom..

[6] Sforza S., Cavatorta V., Lambertini F., Galaverna G., Dossena A., Marchelli R. (2012). Cheese peptidomics: A detailed study on the evolution of the oligopeptide fraction in Parmigiano-Reggiano cheese from curd to 24 months of aging. J. Dairy Sci..

[7] Sieber R., Bütikofer U., Egger C., Portmann R., Walther B., Wechsler D. (2010). ACE-inhibitory activity and ACE-inhibiting peptides in different cheese varieties. Dairy Sci. Technol..

[8] López-Expósito I., Miralles B., Amigo L., Hernández-Ledesma B., Frias J., Martinez-Villaluenga C., Peñas E. (2017). Health effects of cheese components with a focus on bioactive peptides. Fermented Foods in Health and Disease Prevention.

[9] Solieri L., Bianchi A., Giudici P. (2012). Inventory of non-starter lactic acid bacteria from ripened Parmigiano-Reggiano cheese as assessed by a culture dependent multiphasic approach. Syst. Appl. Microbiol..

[10] Bottari B., Levante A., Neviani E., Gatti M. (2018). How the fewest become the greatest. *L. casei*’s impact on long ripened cheeses. Front. Microbiol..

[11] Tagliazucchi D., Baldaccini A., Martini S., Bianchi A., Pizzamiglio V., Solieri L. (2020). Cultivable non-starter lactobacilli from ripened Parmigiano Reggiano cheeses with different salt content and their potential to release anti-hypertensive peptides. Int. J. Food Microbiol..

[12] O’Donnell M., Mente A., Yusuf S. (2015). Sodium intake and cardiovascular health. Circ. Res..

[13] Sacks F.M., Lichtenstein A.H., Wu J.H.Y., Appel L.J., Creager M.A., Kris-Etherton P.M., Miller M., Rimm E.B., Rudel L.L., Robinson J.C. (2017). Dietary fats and cardiovascular disease: A presidential advisory from the American Heart Association. Circulation.

[14] OECD-FAO (2018). Chapter 7. Dairy and dairy products. OECD-FAO Agricultural Outlook 2018–2027.

[15] McCarthy C.M., Kelly P.M., Wilkinson M.G., Guinee T.P. (2017). Effect of fat and salt reduction on the changes in the concentrations of free amino acids and free fatty acids in Cheddar-style cheeses during maturation. J. Food Comp. Anal..

[16] McCarthy C.M., Kelly P.M., Wilkinson M.G., Guinee T.P. (2016). Effect of salt and fat reduction on proteolysis, rheology: And cooking properties of Cheddar cheese. Int. Dairy J..

[17] Møller K.K., Rattray F.P., Bredie W.L.P., Høier E., Ardö Y. (2013). Physicochemical and sensory characterization of Cheddar cheese with variable NaCl levels and equal moisture content. J. Dairy Sci..

[18] Mistry V.V. (2001). Low fat cheese technology. Int. Dairy J..

[19] Guinee T.P., McSweeney P.L.H., O’Mahony S.A. (2016). Protein in cheese products: Structure-function relationships. Advanced Dairy Chemistry.

[20] Wilkinson M.G., Guinee T.P., O’Callaghan D.M., Fox P.F. (1994). Autolysis and proteolysis in different strains of starter bacteria during Cheddar cheese ripening. J. Dairy Res..

[21] Collins Y.F., McSweeney P.L.H., Wilkinson M.G. (2003). Evidence for a relationship between autolysis of starter bacteria and lipolysis in Cheddar cheese. J. Dairy Res..

[22] Fenelon M.A., O’Connor P., Guinee T.P. (2000). The effect of fat content on the microbiology and proteolysis in Cheddar cheese during ripening. J. Dairy Sci..

[23] Tidona F., Bernardi M., Francolino S., Ghiglietti R., Hogenboom J.A., Locci F., Zambrini V., Carminati D., Giraffa G. (2019). The impact of sodium chloride reduction on Grana-type cheese production and quality. J. Dairy Res..

[24] Adler-Nissen J. (1979). Determination of the degree of hydrolysis of food protein hydrolysates by trinitrobenzensulfonic acid. J. Agric. Food Chem..

[25] Re R., Pellegrini N., Proteggente A., Pannala A., Yang M., Rice-Evans C. (1999). Antioxidant activity applying an improved ABTS radical cation decolorization assay. Free Radical Bio. Med..

[26] Rutella G.S., Tagliazucchi D., Solieri L. (2016). Survival and bioactivities of selected probiotic lactobacilli in yogurt fermentation and cold storage: New insight for developing a bi-functional dairy food. Food Microbiol..

[27] Tagliazucchi D., Martini S., Shamsia S., Helal A., Conte A. (2018). Biological activity and peptidomic profile of in vitro digested cow, camel, goat and sheep milk. Int. Dairy J..

[28] Martini S., Conte A., Tagliazucchi D. (2020). Effect of ripening and in vitro digestion on the evolution and fate of bioactive peptides in Parmigiano Reggiano cheese. Int. Dairy J..

[29] Nielsen S.D., Beverly R.L., Qu Y., Dallas D.C. (2017). Milk bioactive peptide database: A comprehensive database of milk protein-derived bioactive peptides and novel visualization. Food Chem..

[30] Grillo A., Salvi L., Coruzzi P., Salvi P., Parati G. (2019). Sodium intake and hypertension. Nutrients.

[31] Briggs M.A., Petersen K.S., Kris-Etherton P.M. (2017). Saturated fatty acids and cardiovascular disease: Replacements for saturated fat to reduce cardiovascular risk. Healthcare.

[32] Pangallo D., Kraková L., Puškárová A., Šoltys K., Bučková M., Koreňová J., Budiš J., Kuchta T. (2019). Transcription activity of lactic acid bacterial proteolysis-related genes during cheese maturation. Food Microbiol..

[33] Schroeder C.L., Bodyfelt F.W., Wyatt C.J., McDaniel M.R. (1988). Reduction of sodium chloride in cheddar cheese: Effect on sensory, microbiological, and chemical properties. J. Dairy Sci..

[34] Kelly M., Fox P.F., McSweeney P.L.H. (1996). Effect of salt-in-moisture on proteolysis in Cheddar-type cheese. Milchwissenschaft.

[35] Rulikowska A., Kilcawley K.N., Doolan I.A., Alonso-Gomez M., Nongonierma A.B., Hannon J.A., Wilkinson M.G. (2013). The impact of reduced sodium chloride content on Cheddar cheese quality. Int. Dairy J..

[36] Baptista D.P., Araújo F.D.S., Eberlin M.N., Gigante M.L. (2017). Reduction of 25% salt in Prato cheese does not affect proteolysis and sensory acceptance. Int. Dairy J..

[37] Piuri M., Sanchez-Rivas C., Ruzal S.M. (2003). Adaptation to high salt in Lactobacillus: Role of peptides and proteolytic enzymes. J. Appl. Microbiol..

[38] Fox P.F., Whitaker J.R., Voragen A.G.J., Wong D.W.S. (2003). Significance of indigenous enzymes in milk and dairy products. Handbook of Food Enzymology.

[39] Summer A., Franceschi P., Formaggioni P., Malacarne M. (2014). Characteristics of raw milk produced by free-stall or tie-stall cattle herds in the Parmigiano-Reggiano cheese production area. Dairy Sci. Technol..

[40] Summer A., Franceschi P., Formaggioni P., Malacarne M. (2015). Influence of milk somatic cell content on Parmigiano-Reggiano cheese yield. J. Dairy Res..

[41] Giraffa G., Mucchetti G., Neviani E. (1996). Interactions among thermophilic lacto-bacilli during growth in cheese whey. J. Appl. Bacteriol..

[42] Giraffa G., Neviani E. (1999). Different *Lactobacillus helveticus* strain populations dominate during Grana Padano cheesemaking. Food Microbiol..

[43] Lombardi A., Maistro L.D., De Dea P., Gatti M., Giraffa G., Neviani E. (2002). A polyphasic approach to highlight genotypic and phenotypic diversities of *Lactobacillus helveticus* strains isolated from dairy starter cultures and cheeses. J. Dairy Res..

[44] Bottari B., Santarelli M., Neviani E., Gatti M. (2010). Natural whey starter for Parmigiano Reggiano: Culture-independent approach. J. Appl. Microbiol..

[45] Coppola R., Nanni M., Iorizo M., Sorrentino A., Sorrentino E., Chiavari C., Grazia L. (2000). Microbiological characteristics of Parmigiano Reggiano cheese during the cheesemaking and the first months of the ripening. Le Lait.

[46] De Dea Lindner J., Bernini V., De Lorentiis A., Pecorari A., Neviani E., Gatti M. (2008). Parmigiano Reggiano cheese: Evolution of cultivable and total lactic micro-flora and peptidase activities during manufacture and ripening. Dairy Sci. Technol..

[47] Bütikofer U., Meyer J., Sieber R., Wechsler D. (2007). Quantification of the angiotensin-converting enzyme inhibiting tripeptides Val-Pro-Pro and Ile-Pro-Pro in hard, semi-hard and soft cheeses. Int. Dairy J..

[48] Uenishi H., Kabuki T., Seto Y., Serizawa A., Nakajima H. (2012). Isolation and identification of casein-derived dipeptidyl-peptidase 4 (DPP-4)-inhibitory peptide LPQNIPPL from gouda-type cheese and its effect on plasma glucose in rats. Int. Dairy J..

[49] Gupta A., Mann B., Kumar R., Sangwan R.B. (2009). Antioxidant capacity of Cheddar cheeses at different stages of ripening. Int. J. Dairy Technol..

[50] Bottesini C., Paolella S., Lambertini F., Galaverna G., Tedeschi T., Dossena A., Marchelli R., Sforza S. (2013). Antioxidant capacity of water soluble extracts from Parmigiano-Reggiano cheese. Int. J. Food Sci. Nutr..

[51] Fleminger G., Ragones H., Merin U., Silanikove N., Leitner G. (2013). Low molecular mass peptides generated by hydrolysis of casein impair rennet coagulation of milk. Int. Dairy J..

[52] Lozo J., Strahinic I., Dalgalarrondo M., Chobert J.M., Haertlé T., Topisirovic C. (2011). Comparative analysis of β-casein proteolysis by PrtP proteinase from *Lactobacillus paracasei* subsp. *paracasei* BGHN14, PrtR proteinase from *Lactobacillus rhamnosus* BGT10 and PrtH proteinase from *Lactobacillus helveticus* BGRA43. Int. Dairy J..

[53] Solieri L., De Vero L., Tagliazucchi D. (2018). Peptidomic study of casein proteolysis in bovine milk by *Lactobacillus casei* PRA205 and *Lactobacillus rhamnosus* PRA331. Int. Dairy J..

[54] Juillard V., Laan H., Kunji E.R.S., Jeronimus-Stratingh C.M., Bruins A.P., Konings W.N. (1995). The extracellular P_I_-type proteinase of *Lactococcus lactis* hydrolyzes β-casein into more than one hundred different oligopeptides. J. Bacteriol..

[55] Maeno M., Yamamoto N., Takano T. (1996). Identification of antihypertensive peptides from casein hydrolysate produced by a proteinase from *Lactobacillus helveticus* CP790. J. Dairy Sci..

[56] Kunda P.B., Benavente F., Catalá-Clariana S., Giménez E., Barbosa J., Sanz-Nebot V. (2012). Identification of bioactive peptides in a functional yogurt by micro liquid chromatography time-of-flight mass spectrometry assisted by retention time prediction. J. Chromatogr. A.

[57] Basiricò L., Catalani E., Morera P., Cattaneo S., Stuknyte M., Bernabucci U., De Noni I., Nardone A. (2015). Release of angiotensin converting enzyme-inhibitor peptides during in vitro gastrointestinal digestion of Parmigiano-Reggiano PDO cheese and their absorption through an in vitro model of intestinal epithelium. J. Dairy Sci..

[58] Stuknyte M., Cattaneo S., Masotti F., De Noni I. (2015). Occurrence and fate of ACE-inhibitor peptides in cheeses and in their digestates following in vitro static gastrointestinal digestion. Food Chem..

[59] Miguel M., Recio I., Ramos M., Delgado M.A., Aleixandre M.A. (2006). Antihypertensive effect of peptides obtained from Enterococcus faecalis-fermented milk in rats. J. Dairy Sci..

[60] Quiros A., Ramos M., Muguerza B., Delgado M.A., Miguel M., Aleixandre A., Recio I. (2007). Identification of novel antihypertensive peptides in milk fermented with *Enterococcus faecalis*. Int. Dairy J..

[61] Robert M.C., Razaname A., Mutter M., Juillerat M.A. (2004). Identification of angiotensin-I-converting enzyme inhibitory peptides derived from sodium caseinate hydrolysates produced by *Lactobacillus helveticus* NCC 2765. J. Agric. Food Chem..

[62] Donkor O.N., Henriksson A., Singh T.K., Vasiljevic T., Shah N.P. (2007). ACE-inhibitory activity of probiotic yoghurt. Int. Dairy J..

[63] Eisele T., Stressler T., Kranz B., Fischer L. (2013). Bioactive peptides generated in an enzyme membrane reactor using *Bacillus lentus* alkaline peptidase. Eur. Food Res. Technol..

[64] Miguel M., Gómez-Ruiz J.A., Recio I., Aleixandre A. (2010). Changes in arterial blood pressure after single oral administration of milk-casein-derived peptides in spontaneously hypertensive rats. Mol. Nutr. Food Res..

[65] Cicero A.F.G., Fogacci F., Colletti A. (2017). Potential role of bioactive peptides in prevention and treatment of chronic diseases: A narrative review. Br. J. Pharmacol..

[66] Korhonen H., Pihlanto-Leppäla A., Rantamäki P., Tupasela T. (1998). Impact of processing on bioactive proteins and peptides. Trends Food Sci. Technol..

[67] Gómez-Ruiz J.Á., Ramos M., Recio I. (2004). Identification and formation of angiotensin converting enzyme-inhibitory peptides in Manchego cheese by high-performance liquid chromatography-tandem mass spectrometry. J. Chromatogr. A.

[68] Meyer J., Bütikofer U., Walther B., Wechsler D., Sieber R. (2009). Changes in angiotensin-converting enzyme inhibition and concentrations of the tripeptides Val-Pro-Pro and Ile-Pro-Pro during ripening of different Swiss cheese varieties. J. Dairy Sci..

